# Digitising psychiatry? Sociotechnical expectations, performative nominalism and biomedical virtue in (digital) psychiatric praxis

**DOI:** 10.1111/1467-9566.12811

**Published:** 2018-09-02

**Authors:** Martyn Pickersgill

**Affiliations:** ^1^ Centre for Biomedicine, Self and Society Edinburgh Medical School University of Edinburgh Edinburgh UK

**Keywords:** E‐health, health technology/technology assessment, mental health and illness, psychiatry/psychiatric care, STS (science and technology studies)

## Abstract

Digital artefacts and infrastructures have been presented as ever more urgent and necessary for mental health research and practice. Telepsychiatry, mHealth, and now digital psychiatry have been promoted in this regard, among other endeavours. Smartphone apps have formed a particular focus of promissory statements regarding the improvement of epistemic and clinical work in psychiatry. This article contextualises and historicises some of these developments. In doing so, I show how purportedly novel fields have been constituted in part through practices of ‘performative nominalism’ (whereby articulations of a neologism in relation to established and recent developments participate in producing the referent of the new term). Central to this has been implicit and explicit extolment of what I term biomedical virtues in public‐facing and professionally orientated discourse. I document how emphases on various virtues have shifted with the attention of psychiatry to different digital modalities, culminating with knowledge‐production in mental health as one significant focus.

## Introduction

Within psychiatry and psychology, digital artefacts and infrastructures have been presented as urgent and necessary. In the UK, for instance, Prime Minister Theresa May promised ‘a £67.7 million digital mental health package’ (HM Government, 2017). Various articles ostensibly focused on the digital within mental health in general or psychiatry specifically attend to heterogeneous (and often mundane) information and communication technologies (ICT) such as text messaging, web sites, blogs, social media and apps (Harding *et al*. 2015, Mohr *et al*. 2017, Torous 2014). Mobile digital technologies have been employed not only as interventions for mental ill‐health, but are on occasion positioned as a means of generating new kinds of information for psychiatrists about patients and pathologies.

Technologies currently in regular operation include therapeutic interactions enabled by videoconferencing (i.e. telepsychiatry), and computer‐based interventions like online cognitive behaviour therapy (CBT) platforms that are advocated by programs such as NHS England's Improving Access to Psychological Therapies (IAPT) initiative. Furthermore, commonly and sometimes enthusiastically used mobile phone applications have proliferated. These often promise to help individuals live with or recover from feelings and experiences associated with psychiatric disorders. Such apps have been argued to widen the ‘possibilities of therapeutic engagement’ (ibid: 262) and ‘recast the spatiality and temporality of healthcare’ (Trnka 2016: 248, see also Fullager *et al*. 2017).

In this article, I contextualise and historicise some of these developments, charting innovation and discussion about telepsychiatry, mHealth and what has more recently been called digital psychiatry. I argue that these purportedly novel fields have been constituted in part through the implicit and explicit extolment of biomedical virtues in promissory statements about them. I demonstrate how emphases on various virtues have shifted with the attention of psychiatry to different digital modalities, culminating with knowledge‐production in mental health as one significant focus.

## Conceptual Background

In this article, I employ the concept of ‘biomedical virtue’ to characterise some of the positive impulses and practices of psychiatry (as understood by practitioners) that are variously framed as being enhanced through, or deactivated by, digital developments. By this term, I do not quite mean ‘medical ethics’ – in either a straightforwardly moralistic way, or in Osborne's (1994) more capacious definition – nor what Freidson (1988[1970]: 160) called the ‘operative norms of performance’ within medicine. Instead, biomedical virtue refers to the (profession‐defined) praxis of goodness within the laboratory and the clinic. Through widespread instantiation across a range of registers (e.g. public‐facing documents and intra‐professional conversations), I suggest that articulations of virtue might exert disciplining effects on actual practices. Consequently, biomedical virtue can have an ambiguous existence as both aspiration and actuality.

The rhetoric of biomedical virtue can be strategically and more spontaneously deployed in support of various professional projects and goals. This is particularly evident when what is perhaps the canonical biomedical virtue, the prevention of death, is used to justify continued symbolic and material investment in technological processes that are yet to demonstrate clinical worth. Martin *et al*. (2008) provide a striking example of this through a case study of private umbilical cord blood stem cell banking. They demonstrate how a market has emerged, underpinned by visions of cord blood as having *potential* therapeutic application for assorted life‐limiting or threatening disorders that a new‐born baby might one day experience. Accordingly, the biomedical virtue of preventing death (or at least, severe suffering) has played a key role in powering a promissory bioeconomy (ibid). More generally, diverse forms of therapeutic promise (Rubin 2008: 13) have been constituted with and through various kinds of biomaterials, configuring these as promissory matter (Brown *et al*. 2006: 330) to which hopes and capital are associated. In effect, articulating virtue can generate value.

Professional projects that might involve the strategic deployment of biomedical virtues to justify their necessity include practices of what I call ‘performative nominalism’. Intellectual debts are owed here to Ian Hacking, whose concept of dynamic nominalism captures how, sometimes, ‘our classifications and our cases emerge hand in hand, each egging the other one’ (Hacking 2002: 106). My adaptation of Hacking's term underscores the role of agency: co‐emergence can be deliberately and actively fostered by agents who reflexively reconfigure their (e.g. professional) identities in the process. Practices reminiscent of performative nominalism come from a variety of fields; sociologists of biomedical ethics, for instance, have demonstrated how ethical commentary shapes technoscientific development and thus the need and role of bioethicists (Hedgecoe and Martin 2003). Closer both to psychiatry and to my specific framing of performative nominalism, scholars like Brosnan (2011) and Conrad and De Vries (2011) have detailed how the deployment of the neologism ‘neuroethics’ has contributed to the organisation of a field and to consolidating its authority.

Such insights emerge from wider work on the sociology of expectations, and form a more general backdrop to the analysis presented here. As Brown and Michael (2003: 3) argue, expectations ‘are crucial to providing the dynamism and momentum upon which so many ventures in science and technology depend’ (see also Borup *et al*. 2006, Brown 2003). In the case of biomedicine, the promise of new health technologies involves the mobilisation of ‘a range of claims about their future therapeutic impact’ (Webster 2002: 443). This includes psychiatry, where investments in neuroscience, for instance, are predicated in part upon the promise that these will enhance clinical practice (Pickersgill 2011). In what follows, I regard performative nominalism as one mechanism within the sociology of expectations, and biomedical virtues as legitimising tropes for promises made. As we will see, promissory discourse has been a key feature of commentary upon and pronouncements regarding the role and impact of digital technologies in and on psychiatry.

## Methodological Approach

This article follows a similar methodological approach to Williams *et al*.'s (2015) analysis of trends and transformations in the area of digital interventions for monitoring and managing sleep. Specifically, I interrogated various (online) documents to ascertain how ‘problems, prospects and possibilities’ (Williams *et al*. 2015, : 1041) relating to the use of mobile digital technologies psychiatry were expressed. I was particularly attentive to what had come to count as ‘digital psychiatry’, how this discourse was being propelled, and what its antecedents were. In so doing, I was alert to ‘uncertainty statements’ (Webster 2002: 443); that is, comments suggestive of an organised ambivalence (cf. Benjamin 2011) within psychiatry that simultaneously embraces hope for innovation while recognising the potential for disappointment.

Some of the texts discussed in this article were initially collected and examined as part of three Wellcome Trust projects on the sociology of mental health. The first, which took place over 2011–2015, considered how the need for enhancing access to mental healthcare had emerged as a political and clinical concern in the UK, how it was being addressed, and what the ramifications of change were for clinicians and patients. The second study, begun in 2015 and ongoing, analyses how ideas about the nature of diagnosis in US and UK psychiatric research and practice are moving in response to various challenges within and beyond the mental health disciplines. Initiated in 2017, the third project forms part of a wider programme of work on ‘Biomedicine, Self and Society’. Through this, I am exploring shifting logics of neuroscience and mental health that migrate away from an exclusive focus on psychopathology and extend into the study of ‘normal’ subjective experience.

As part of the continuing documentary analysis, interviewing, focus groups and observation at clinical symposia that comprises these projects, I realised I was encountering seemingly increasing – and increasingly high profile – references to digital mental health technologies. Discussions of apps, for instance, were found within government agenda‐setting documents, research funder roadmaps and commentaries in high‐impact psychiatry journals. The data corpus for this article thus developed from these materials, and was augmented through additional targeted searches in key journals and websites such as that of the UK Department of Health and the American Psychiatric Association (as well as through examining links between the aforementioned documents and other relevant commentaries and position statements). My broadly abductive (Timmermans and Tavory 2012) analysis accordingly entailed both a close reading of isolated texts referring to digital mental health, and a consideration of these in relation to the data collected within the aforementioned studies, and the larger problematic they address: that is, in what ways is mental health praxis changing, and what is powering those shifts?

## Psychiatry and Technology

What greater scourge could befall psychiatry than becoming impersonal – which means losing sight of the persona of the patient? The great technological advances that have taken place in medicine within the last three‐quarter century raise this threat – the loss of the personal relationship with the patient. The whole tradition is based on healing and caring for the sick as persons, through constant personal contact between the doctor and the patient (Bartemeier 1952: 1)

The comments above come from Leo H. Bartemeier's 1952 Presidential Address to the American Psychiatric Association (APA). He used this to sound caution at the rise of techn(olog)ical approaches to clinical practice, and to underscore the therapeutic import of interpersonal relationships. Bartemeier's anxieties orientate us to the great degree to which psychiatrists have long engaged with a wide range of technologies. These often emerged from within allied disciplines such as neurology (e.g. EEG; Schirmann 2014), physiology (e.g. PET; Dumit 2004) and nuclear medicine (e.g. MRI; Joyce 2008). In the mid‐20^th^‐century US, psychiatrists like Bartemeier were often highly psychoanalytically orientated, (Hale 1995); yet, interventions into neurological structure and function to treat mental ill‐health were widely employed. Most controversially, these included ECT and lobotomy. Sadowsky (2006: 22) has illuminated how receptive many psychoanalysts were to ECT, underscoring ‘the extent of eclecticism in American psychiatry’. For lobotomy, Raz (2008: 387) demonstrated that ‘psychosurgical discourse adopted key concepts from psychoanalytical discourse’ and vice versa. Hence, technical apparatuses within psychiatry can be understood to help direct practitioner attention, while also becoming enfolded within existing theoretical and operational regimes (Pickersgill 2010).

‘Telepsychiatry’ is a more contemporary instantiation of a (partial) psychiatric embracement of an external technological innovation; namely, telemedicine (APA, 2017b). Now a heterogeneous discipline encompassing an array of technologies and applications (Lupton and Maslen 2017), this was defined in the first editorial of the Telemedicine Journal as: ‘the delivery of care to patients anywhere in the world by combining communications technology with medical expertise’ (Goldberg 1995: 1). For almost 30 years, clinicians and policymakers have advocated telemedicine as a means of containing healthcare costs and enhancing access (Lehous *et al*. 2002). In the UK, for instance, early policy announcements emphasised ‘the novelty and potential of telemedicine systems in a rhetoric that stresses the technology's place in a paradigm shift in the conceptualization and organization of British health care’ (May *et al*. 2001: 1890). It was in part through such discourse, and via journals, research groups, professional associations and conferences that bore the name ‘telemedicine’, that the field was constituted (ibid: 1981). This can be regarded as a form of performative nominalism: the coining of ‘telemedicine’ and the badging of activities as such contributed to galvanising hopes and resources in ways that facilitated its consolidation.

Telepsychiatry is defined straightforwardly by organisations like the APA as an operation whereby ‘psychiatric services [are] provided remotely via a video link’ (APA, 2017b), and the Royal Australian & New Zealand College of Psychiatrists (RANZCP) as ‘a consultation between a patient and a psychiatrist conducted via video‐conference’ (RANZCP, undated). These professional bodies suggest that specific uses of telepsychiatry include ‘psychiatric evaluations, therapy (individual therapy, group therapy, family therapy), patient education and medication management’ (APA, 2017b; see, similarly, RANZCP, 2013: 5). Telepsychiatry apparently ‘offers many advantages and is becoming more available’ (APA, 2017b); as current APA President Anita Everett stated, one such advantage includes the use of telepsychiatry to ‘help increase access’ to care (APA, 2017a; see also Bender 2008). Similarly, the RANZCP website poses the question, ‘Why use telepsychiatry?’, and goes on to answer that it ‘can greatly improve access to psychiatric services for people in rural and remote areas, and in other situations where face‐to‐face consultations are impracticable’ (RANZCP, undated).

More generally, research and practice initiatives around telepsychiatry in a range of nations have been inscribed (Akrich 1992) with this logic of access; intervention trials, for instance, commonly note the utility of the technology in terms of enhancing admission into therapy for those who might otherwise be excluded from care (e.g. Kessler *et al*. 2009, Nelson *et al*. 2003, O'Reilly *et al*. 2007).^1^ Clinicians, though, have often been presented as resistant to the introduction of telepsychiatry due to assumed deleterious effects on the relationship between doctor and patient deemed central to therapeutic engagement and success. As May *et al*. (2001) and (Wagnild *et al*. 2006) have shown, such matters have inflected the reception of telepsychiatry – and telemedicine more generally (May *et al*. 2003) – for two decades now, with the technological shaping of inter‐subjective action constitutive of telepsychiatric practice considered an object of clinical concern.

Telepsychiatry, then, has been constructed as simultaneously enabling and curtailing different kinds of biomedical virtue in psychiatry. On the one hand, and most importantly within promissory discourse, telepsychiatry might increase access to therapy. Even half a century ago, access had been – in the words of APA Medical Director Walter Barton – ‘a concern of the APA for a long time’ (Barton 1971: 522). Today, the enhancement of access continues to be a virtue (co‐)constituted through a range of (inter)national policy and clinical debates. These are underpinned by a form of humanitarian reason (Fassin 2011) in which access is configured as an unproblematic social good. On the other hand, telepsychiatry – as May *et al*. (2001) and Wagnild *et al*. (2006) have documented, and as I have sometimes encountered in my wider fieldwork – is judged to have the potential to undercut a positive doctor–patient relationship. The degree to which the development, maintenance and promotion of this dynamic is felt by many to be virtuous is apparent through recurrent celebrations of the doctor–patient relationship as (for instance) ‘a keystone of care’ (Goold and Lipkin 1999: S26). The import of this virtue is also illuminated by a wide‐ranging literature describing various purported challenges to it, including industrial, economic and political impacts on healthcare systems. Perhaps, especially significant in psychiatry, given the role of inter‐subjectivity *as* therapy, the doctor‐patient relationship is recurrently underscored as of ‘central importance’ (as Allan Tasman put it during an APA Presidential Address) (Tasman 2000: 1762). In the case of psychiatry, then, discourse upon the use of communicative technologies within the broad field of telepsychiatry directs attention to two different modes of biomedical virtue, one of which may interfere (Pols 2003) with the other.

## ‘Mobilising’ Psychiatry

In the previous section, I introduced some of the ways psychiatry has negotiated new biomedical technologies, focussing in particular on telepsychiatry. This has been a major focus for psychiatric thought about how to interweave information and communications technologies into clinical practice, as well as a striking example of how particular biomedical virtues configure field‐building and the reception of innovation. It is not, of course, the only illustration that could be advanced. Online psychological therapy, in particular CBT, has likewise been advocated and debated, with a range of articles appearing in widely read periodicals (e.g. Psychiatric News and the American Journal of Psychiatry). In the UK, online therapy portals like Beating the Blues have been rolled out across the NHS, and are endorsed by bodies such as the National Institute of Health and Care Excellence (NICE) in England and the Scottish Intercollegiate Guidelines Network (SIGN). Online therapies, where users navigate through pre‐set text and exercises, tend to be associated with the treatment of so‐called ‘mild to moderate’ instantiations of common conditions like depression, and are deployed through psychological and primary care services. As well as pre‐set content, newer tools using chatbots are more interactive – like ‘woebot’, a ‘fully automated conversational agent’ (Fitzpatrick *et al*. 2017). Considerations of telepsychiatric approaches as avenues for treatment vary more widely across a range of conditions, including those for which psychiatrists often take clinical responsibility (e.g. schizophrenia). In recent years, telepsychiatry has become frequently operationalised through mobile phones, and online therapies – once accessed largely through desktop PCs – have been ‘mobilised’ as smartphone applications (i.e. apps).

In this section, I explore how apps are being promoted through the diverse field of ‘mHealth’ (or ‘m‐health’; i.e. ‘mobile health’). As sociologists Williams, Coveney and Meadows have discussed, through mobile digital technologies ‘patients, proto‐patients and the wider public may now track their bodies, share their data, participate in online discussion and support groups, and use the information they gather to improve or optimise their health’ (Williams *et al*. 2015: 1045). mHealth is a key facet of this matrix of illness, optimisation and technology. In one public‐facing article, the UK Medical Research Council (MRC) defined mHealth as ‘the name for medicine and public health supported by mobile phones; now a burgeoning field’ (MRC, 2015). According to the APA (undated), the ‘expanding use of mobile health (mHealth) technologies is unprecedented in the history of medicine’ – and there has been ‘growing patient, clinical, government, and payer interest in the potential of mHealth technologies for psychiatric clinical care’. To this end, the APA convened a working group to interrogate mobile phone apps which assert that they promote wellbeing and treat mental ill‐health. The group went on to develop an evaluation model for psychiatrists to use when ascertaining whether an app might afford a patient therapeutic benefit.

One key difference between the deployment of telepsychiatry and mental health apps is the responsibilisation of the user. In the former case, the onus lies with the clinician to learn and implement new skills and care practices; in the latter, the responsibility lies primarily (though as we will later see, not exclusively) with the purportedly empowered patient. For instance, the World Health Organization recommended in their Mental Health Action Plan that ‘the promotion of self‐care’ for mental ill‐health could be encouraged through ‘the use of electronic and mobile health technologies’ (WHO 2013: 14). In the UK, the MRC (2015) has argued that mHealth ‘offers a simple and low cost way to empower patients to take responsibility for their own health – something that's particularly effective in mental health’.

The innovation and implementation of mHealth within the UK has been supported by a strong policy push towards innovation (HM Government, 2017; Mental Health Taskforce, 2016: 38; National Information Board, 2014). Investment has come, for instance, from the NHS National Institute for Health Research, such as in the MindTech Healthcare Technology Co‐operative: ‘a national centre focussing on the development, adoption and evaluation of new technologies for mental healthcare and dementia’.^2^ Indeed, the NHS hosts an online library of apps where patients are invited to ‘Find digital tools to help you manage and improve your health’ (NHS, 2018). In August 2018, 19 apps featured under the ‘Mental Health’ section of the site, three of which were badged as ‘Being Tested in the NHS’. One of these, Chill Panda, carries the strapline, ‘Learn to relax, manage your worries and improve your wellbeing with Chill Panda’. Nevertheless, a disclaimer notes that apps in the Library ‘are not intended to be a substitute for a consultation with a healthcare professional. It is up to you to contact a healthcare professional if you are concerned about your health’ (NHS, 2018). Clinical expertise thus remains salient, even as (potential) patients are encouraged to contribute to their care.

In the US, the National Institute of Mental Health (NIMH) has, like other sponsors internationally, actively fostered research into mHealth in part through promissory statements articulating tropes of biomedical virtue. It was, for instance, explicitly flagged within the agency's Strategic Plan for Research. This 60‐page document described four Strategic Objectives for the NIMH, each of which included two ‘Highlights’ which contextualised the Objectives through details of existing and anticipated research. The final Highlight – ‘A Therapist in One's Pocket: mHealth to Improve Access to Mental Health Care’ – posited digital technologies ‘such as smartphones, wearable sensors, and video games’ as having the capacity to address ongoing problems around access to healthcare. (NIMH, 2015: 51). Like telepsychiatry before it, then, mHealth was thus presented as a technological fix to an issue of healthcare policy, planning and delivery. Nevertheless, the biomedical virtue of direct clinical care was inscribed into the Highlight, with the NIMH making clear that digital tools will ‘extend rather than replace the therapist’ – at least for ‘those with the most disabling illnesses’ (ibid). As for specific uses, mobile technologies were described as being employed currently ‘for improving adherence to treatment or for collecting passive data about activity or sleep, but the additional possibilities of these technologies are just emerging’ (NIMH, 2015: 51). Such entangling of the actual and the anticipatory lends credibility to the promise underpinning the forward‐looking statements threading through the Highlight.

More recently, a NIH‐wide Funding Opportunity Announcement (FOA; posted 30 November 2017) encouraged applications that ‘develop or adapt innovative mobile health (mHealth) technology specifically suited for low‐ and middle‐income countries (LMICs) and determine the health‐related outcomes associated with implementation of the technology’ (NIH, 2017). The specifically NIMH component of the FOA called for the ‘development or innovative application of cost‐effective, sustainable, and scalable technologies to improve the accessibility, effectiveness, or delivery of mental health care in LMICs’. Similarly, the MRC (2015) has noted that ‘the popularity of mobile phones isn't confined to developed countries. Their widespread use, even in the most remote locations, could help people get faster and easier access to healthcare’. Such calls and statements from funders like the NIMH and the MRC underscore how mHealth, including for psychiatric intervention, is being folded into highly financed regimes of what has been termed ‘global health’ (as intimated by Williams *et al*. 2015: 1051).^3^

One means of tracking the evolution and increasing visibility of mHealth is through the statements made about it by the psychiatrist and former NIMH Director Thomas Insel (in post 2002–2015). Once cautious about the use of technological interventions in mental health (Shoham and Insel 2011), Insel has become an ever more vocal advocate of (mobile) digital techniques in psychiatric settings. When, during a 2013 *Boston Globe* interview, he was queried about the future of psychotherapy, Insel described how it ‘might be that what we call “psychotherapy” is a mobile app that you download and is crafted for your specific cognitive domain’ (Koven 2013). A year later, *Psychology Today* asked Insel ‘What's a clinical innovation you're excited about?’; he responded: ‘Web‐based and mobile CBT’ (Nemko 2014). The following March, Insel was quoted in *Counseling Today* describing how the ‘advent of new devices, mHealth (mobile health), cognitive training and social media will likely change the landscape of mental health care’ (Burnett 2015). In 2015, Insel left the NIMH to work for Alphabet on device‐orientated approaches to mental health. In a discussion with *Fortune* magazine, he accounted for this interest in technology as relating to wider shifts in understandings of psychopathology: where once disorders like depression were seen as ‘just a chemical imbalance’, now they are considered to be ‘problems with how neural circuits are communicating’ – an issues that ‘we may be able to modulate with devices’ (Sukel 2015).

Despite a widespread lauding of mHealth, concerns have also been raised about the security and commodification of the data that apps might harness, given their commonly commercial origins (Lupton 2014). This includes mental health apps (Nicholas *et al*. 2015), with one news piece for instance addressing the problem explicitly and bluntly: ‘Many app developers may be selling patient data for profit’ (Torous *et al*. 2016). In a review of opportunities and challenges associated with apps, psychiatrists Marley and Farooq (2015) also highlighted concerns about the clinical accuracy of information presented, and the (lack of a) role of medical expertise in the development of some software – as well as overt risk to patients using apps partly as a consequence of errors and omissions (see also Zhang *et al*. 2015). In an analysis of the challenges associated with digital technologies and mental health, Ben‐Zeev (2017: 108) argued that the ‘client‐clinician therapeutic alliance will be affected by the introduction of telecommunication and telemonitoring technology’. The discussions about the hazards of mHealth proliferating within psychiatric discourse underscore the increasingly everyday nature of mobile digital technologies for mental health, as well as the responsibility many clinicians feel regarding the need to better understand the risks and benefits associated with them when negotiating patients’ own questions and concerns.

Through disseminating and defining the concept of mHealth, and generating ethical discourse around its instantiations, professional associations like the APA and funders like the MRC and NIMH contribute to processes of performative nominalism, helping to reify mHealth as an object of (cautious) fascination. Within (for instance) the concerns of physicians, the disclaimers of NHS websites, and the tacit and overt endorsement of mental health apps by health‐related organisation, we can see at least two biomedical virtues (beyond the enhancement of access) as playing a role in this performative nominalism. For one, there is – as explicitly stated by the WHO (2013: 14) – the virtue of encouraging self‐care. As Rose (2001), among others, has pointed out, public health in a range of countries commonly emphasises care of the self in order to stave off pathology and optimise present health. Self‐care is increasingly presented as a virtue, with clinical professionals and societies framing it as a means of empowering patients and cutting healthcare costs (see Rimmer (2014) for a striking example).

Second, we can see the virtue of clinical responsibility. When the NIMH (2015: 51) advances statements that mHealth should ‘extend rather than replace the therapist’, and when the apps recommended by the NHS carry with them a disclaimer that they are not a substitute for a medical consultation, we can see how the ultimate responsibility for defining and treating mental ill‐health is apportioned to a clinician. While articulations of clinical responsibility are necessarily demarcations of power and expertise, they are also configured as virtuous in legalistic healthcare contexts where responsibility is inscribed in hard and soft law. Hence, the formal holding of clinical responsibility, and the performance of being a *responsible* clinician (in a broader sense), are mutually reinforcing and blur the lines between descriptive and normative dimensions of responsibility. Through reminders to patients and professionals within writings on mHealth that mobile technologies should not be substituted for clinical responsibility, the virtue of responsibility comes to legitimise endeavours to innovate the development and implementation of digital devices.

## Consolidating ‘Digital Psychiatry’?

The year 2017 saw the publication of The World Psychiatric Association‐Lancet Psychiatry Commission on the Future of Psychiatry: a report ‘intended to stimulate thought, debate, and the change necessary for psychiatry to fulfil its potential as an innovative, effective, and inclusive medical specialty in the 21st century’ (Bhugra *et al*. 2017: 776). An accompanying editorial highlighted ‘the apparently relentless progress of digital technology’, which has ‘the potential to render physical distance irrelevant to some areas of psychiatric practice’ (Anon, 2017: 733). This creates a new ‘challenge’ to psychiatry ‘to use such innovations to enhance, rather than replace, the humane and humanistic aspects of practice’ (ibid). Similar comments about progress and risks were made in the report Executive Summary:

Digital technology might offer psychiatry the potential for radical change in terms of service delivery and the development of new treatments. However, it also carries the risk of commercialised, unproven treatments entering the medical marketplace with detrimental effect. Novel research methods, transparency standards, clinical evidence, and care delivery models must be created in collaboration with a wide range of stakeholders. Psychiatrists need to remain up to date and educated in the evolving digital world. (Bhugra *et al*. 2017: 775)

The Commission comprised six key sections, one of which was a segment specifically on ‘digital psychiatry’. This level of attention to the role of digital technology within mental health research and practice is indicative of a wider growth in prominence that, as we have seen, has been constituted through political position statements, funding policy and outcomes, and research articles and commentaries. What comprises ‘digital psychiatry’ in the Commission, as elsewhere, is wide‐ranging: smartphones, virtual reality and machine learning are all included. Smartphones, as in many writings on mHealth, are given primary prominence; in the Commission, discussion of apps is juxtaposed with more futuristic possibilities such as augmented reality glasses which are ‘entering the mental health space’ (Bhugra *et al*. 2017: 799), albeit in somewhat undefined ways. Through talk of apps, and of more advanced technologies that ‘are already projected to change health care’ (Bhugra *et al*. 2017: 799), digital psychiatry can be presented as moving ‘beyond traditional telepsychiatry’ (ibid: 798) – and hence requiring new and focused attention and debate.

As Hedgecoe (2010: 172) has demonstrated in pharmacogenetics, ‘scientific review papers bolster expectations’ that new technologies ‘will come into everyday clinical use’. The Commission and other writings reviewing the state of digital psychiatry likewise point to the apparent inevitability of innovation and implementation. One recurrent figure within contemporary writings on technology and mental health is US psychiatrist John Torous. Digital Psychiatry Editor for the Psychiatric Times, Chair of the APA Smartphone App Evaluation Task Force and a co‐author of the WPA‐Lancet Psychiatry Commission, Torous can be regarded as actively constituting a dedicated field of digital psychiatry through documenting its possibilities. This includes articles examining the ethical hurdles associated with the use of digital technologies in mental health, and providing provisional roadmaps for navigating these (e.g. Torous *et al*. 2014, Torous and Nebeker 2017). Through raising (and sometimes resolving) ethical issues, Torous and others contribute to performing the novelty and import of digital innovation in psychiatric praxis. ‘Digital psychiatry’, then, and ‘digital mental health’ more generally, appears not so much as a relatively bounded site of sociotechnical practice, but rather manifests through often promissory discourse about the rise of information and communication technologies and their ramifications for research and therapeutic interventions (cf. Brown 2003, Hedgecoe and Martin 2003).

According to the WPA‐Lancet Psychiatry Commission report, the ‘digital psychiatry revolution has arrived’ (Bhugra *et al*. 2017: 798). As indicated above, this includes smartphone apps to treat mental disorders – but also highlighted in the report, and elsewhere, is the possibility of enrolling these as sources of data for research into the nature of psychopathology. As Dror Ben‐Zeev (2017: 107) wrote in the first column of his new series in Psychiatric Services on technology and mental health: ‘Advancements in Web, mobile, sensor, and informatics technology can do more than serve as tools to enhance existing models of care. Novel technologies can help us better understand the very nature of mental illness’. With similar optimism, the NIMH noted on one of their public‐facing pages how receiving ‘information from a large number of individuals at the same time can increase researchers’ understanding of mental health and help them develop better interventions’ (NIMH, 2017).

The direct data that people themselves enter into apps is described in the Commission report as one example of this. Other writing discussing the use of mobile technologies to enhance knowledge‐production in psychiatry has emphasised, for instance, the collection of ‘data on health behaviours and symptoms in real time and outside of the clinic setting, using text messages’ (Vahia and Depp in Jeste 2013). With regards to self‐inputted data, new industry‐academic partnerships are emerging: for example, between the mental health social networking app TalkLife, Microsoft Research, MIT and Harvard University, which are using machine learning to ‘better understand and predict self harm’ (TalkLife, 2018). The Commission also noted the importance of passive data obtained via some of the additional features of smartphones (like GPS) (Bhugra *et al*. 2017: 799). Other articles and commentaries have similarly flagged this, with sleep‐ and activity‐tracking highlighted as means of generating new temporally fine‐grained insights into psychopathology as experienced in everyday life (e.g. Marzano *et al*. 2015). According to former NIMH Director Thomas Insel, such ‘digital phenotyping’ is already ‘revealing new aspects of behaviour that appear clinically relevant’ (Insel 2017: 1217). Threading through all these commentaries is the biomedical virtue of the enhancement of clinical wisdom: digital technologies might impart new means of comprehending mental ill‐health, and hence of understanding individual patients.^4^

## Conclusion

Innovations in digital mental health promotion and treatment have generated particular excitement internationally (e.g. Hollis *et al*. 2015, Miner *et al*. 2017, Torous 2016) – with one corollary of this being concern and disquiet (Ennis *et al*. 2012, Hayes *et al*. 2016). Through interrogating telepsychiatry, m‐health, and digital psychiatry, I have sought to historicise and enrich contextual understandings of digital developments in mental health. We have seen how, in each of these cases, current uses and future possibilities are connected in ways that imply the inevitability and desirability of innovation.

Through discussions of probable and potential sociotechnical scenarios emerging from commentaries on digital psychiatry and the like, some organisations and individuals adopt roles as ‘mediators of hope’ (Martin *et al*. 2008: 127) regarding new technologies. Of these, agencies like the NIMH have the economic capacity to materialise a selection of the innovations they anticipate and the discourses they promote. Strategies of field‐building are consequently occurring with and through concrete investment and change and seem likely to be playing out with a view to propelling such expenditure and innovation. Some of these strategies can be characterised as practices of what I have termed performative nominalism (following Hacking (2002)). In this respect, purported members of ostensibly new fields act, in effect, to talk these into existence. Through mirroring such rhetoric (e.g. about mHealth), funders and governments themselves contribute to this consolidation.

Within the promissory statements that are so important to the constitution of new endeavours, the implicit and explicit extolment of what I call biomedical virtues plays an important legitimising function. Emphases on various virtues have shifted over time, as the attention of psychiatry has ranged around different digital modalities. To date, the promotion of access to treatment has been a key virtue propelling technological innovation in mental health (as, more recently, has the encouragement of self‐care). Today, mobile technologies are also now being folded into new regimes of psychiatric data collection, constituted through the virtue of the enhancement of clinical wisdom. In particular, service‐orientated research is being conducted and urged, to ascertain the potential for information gleaned from mobile phones (including via apps, but also through activity‐monitoring functions) to assist with clinical decision‐making. This work has been argued to have the potential to reshape therapeutic encounters, whereby the epistemic salience of patient testimony regarding their subjective experience is dissipated as clinically relevant objectivity is delegated to devices. Investigations are also emerging into how digital technologies might provide new insights into the nature (especially, the temporalities) of symptoms like low‐mood. Research in this vein resonates with wider epistemological shifts in psychiatry into the analysis of symptoms and mechanisms of psychopathology (Pickersgill 2014, Rose, 2016), which have (again) rendered problematic the existence of established nosological categories like schizophrenia.

Interfering (Pols 2003) in all this is another biomedical virtue: the maintenance of a positive doctor–patient relationship. As one commentary recently asserted, this relationship is of ‘singular importance’ in psychiatry (Steingard 2018: 1). It is striking how much the editorial accompanying the WPA‐Lancet Psychiatry Commission report, which advanced concerns about the ‘challenge’ of innovation for ‘humane and humanistic aspects of practice’ (Anon, 2017: 733), echoes the worries of psychiatrists in decades past. In particular, Leo Bartemeier's anxieties about the ‘threat’ of technology to the psychiatrists’ ‘personal relationship with the patient’, set out 65 years ago in his APA Presidential Address, seem to be little changed. It thus remains unclear how straightforwardly psychiatrists will accommodate much‐vaunted digital developments in their practice. Nevertheless, based on the eclecticism and pragmatism of psychiatry that I illustrated earlier in this analysis, we might reasonably expect more accommodations than the aforementioned concerns might suggest. Regardless, the normative dimensions of (any) new digital instantiations within psychiatric praxis will certainly continue to bear sociological scrutiny.

## Notes

1 See also Hubley *et al*. (2016) for a review of trials which foregrounds the import of access.

2 See: http://www.mindtech.org.uk.

3 A field that itself could be regarded as consolidated in large part through its naming.

4 One corollary of this is a potential intensification of medicalisation. As Insel's comments imply, practices engaged in by individuals categorised with a psychiatric disorder could be (increasingly) associated with that psychopathology by virtue of the correlational data collected by digital devices.
